# Molecular Pathways and Potential Therapeutic Targets of Refractory Asthma

**DOI:** 10.3390/biology13080583

**Published:** 2024-08-01

**Authors:** Leah Ishmael, Thomas Casale, Juan Carlos Cardet

**Affiliations:** 1 Division of Pulmonary, Allergy, and Sleep Medicine, Mayo Clinic, Jacksonville, FL 32224, USA; ishmael.leah@mayo.edu; 2 Division of Allergy and Immunology, Department of Internal Medicine, University of South Florida Morsani College of Medicine, Tampa, FL 33612, USA; tbcasale@usf.edu

**Keywords:** cytokines, T2 inflammation, non-T2 inflammation, asthma, eosinophils

## Abstract

**Simple Summary:**

Refractory asthma is a complex and heterogeneous disease. The molecular pathways and cells involved in refractory asthma include T2 and non-T2 inflammation, eosinophils, neutrophils, mast cells, and macrophages, among others. This review analyzes the inflammatory and immune dysfunction caused by mediators including cytokines IL-1, IL-6, and TNF-alpha, JAK kinases, and alarmins such as IL-33. Targeting these mediators could potentially offer new therapeutic options for refractory asthma.

**Abstract:**

Asthma is a chronic inflammatory lung disease. Refractory asthma poses a significant challenge in management due to its resistance to standard therapies. Key molecular pathways of refractory asthma include T2 inflammation mediated by Th2 and ILC2 cells, eosinophils, and cytokines including IL-4, IL-5, and IL-13. Additionally, non-T2 mechanisms involving neutrophils, macrophages, IL-1, IL-6, and IL-17 mediate a corticosteroid resistant phenotype. Mediators including alarmins (IL-25, IL-33, TSLP) and OX40L have overlap between T2 and non-T2 inflammation and may signify unique pathways of asthma inflammation. Therapies that target these pathways and mediators have proven to be effective in reducing exacerbations and improving lung function in subsets of severe asthma patients. However, there are patients with severe asthma who do not respond to approved therapies. Small molecule inhibitors, such as JAK-inhibitors, and monoclonal antibodies targeting mast cells, IL-1, IL-6, IL-33, TNFα, and OX40L are under investigation for their potential to modulate inflammation involved in refractory asthma. Understanding refractory asthma heterogeneity and identifying mediators involved are essential in developing therapeutic interventions for patients unresponsive to currently approved biologics. Further investigation is needed to develop personalized treatments based on these molecular insights to potentially offer more effective treatments for this complex disease.

## 1. Introduction

Asthma is a chronic airway disease that affects children and adults. Approximately 5 to 10 percent of patients with asthma have severe or refractory disease characterized by persistent respiratory symptoms despite adherence to medication [1]. The European Respiratory Society and the American Thoracic Society define severe asthma as asthma requiring treatment with high-dose inhaled corticosteroids (ICS) plus a second controller and/or systemic corticosteroids to prevent it from becoming uncontrolled, or that remains uncontrolled despite this therapy. Difficult-to-treat asthma is asthma that is poorly controlled despite therapy because of potentially modifiable factors such as poor medication adherence or environmental exposures and comorbidities such as severe sinus disease [2].

**Figure 1:**
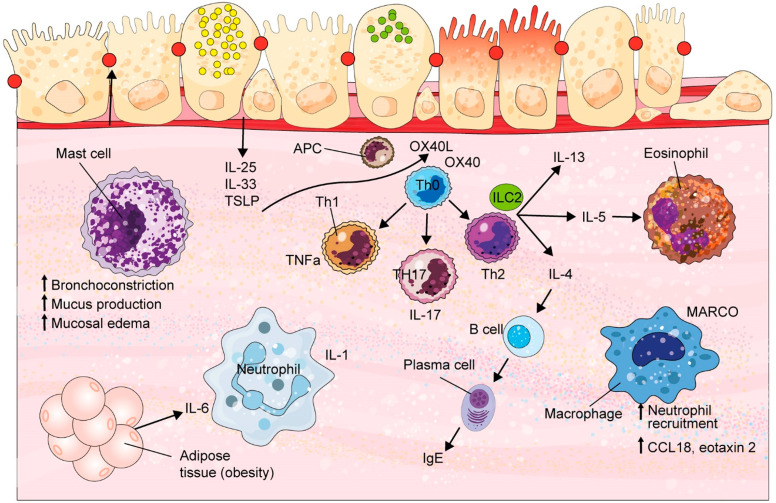
Key signaling pathways and mediators and their role in refractory asthma. APC = antigen-presenting cell; IgE = immunoglobulin E; IL = interleukin; ILC2 = innate lymphoid type 2 cell, MARCO = Macrophage Receptor with Collagenous structure; Th = T helper; TSLP = thymic stromal lymphopoietin; TNFα = tumor necrosis factor alpha.

Poor asthma control is associated with significant morbidity and mortality and increased healthcare utilization [3]. Patients with severe asthma exhibit a variety of physiological characteristics, disease presentations, and responses to available therapeutics. The heterogeneity of severe asthma makes it particularly difficult to treat uniformly. Cohorts such as the Severe Asthma Research Program (SARP) and Unbiased Biomarkers for the Prediction of Respiratory Disease Outcomes (U-BIOPRED) have evaluated clusters of patients with severe asthma using methods such as whole-genome sequencing, transcriptomics, proteomics, and metabolomics in attempts to understand pathophysiologic mechanisms and uncover different subtypes of severe asthma. With a better understanding of the pathobiological pathways involved in severe, refractory asthma, there is hope in finding better and more personalized therapeutic options. In this article, we will review some of the cells, mediators, signaling pathways, and other features of the mechanisms underlying refractory asthma, including those with therapeutic potential. These include eosinophils, mast cells, macrophages, neutrophils, T helper cell subsets, interleukin (IL)-1 receptor signaling, alarmins, IL6, C-C motif chemokine ligand 5 (CCL5), regulators of G protein signaling (RGS), and the microbiome (Figure 1).

## 2. Immune Cells

### 2.1. Eosinophils

Eosinophilic airway inflammation is the hallmark of the most common asthma phenotype and is primarily driven by type 2 (T2) inflammation. T2 inflammation is defined by the involvement of cytokines such as IL-4, IL-5, IL-13, and granulocyte–macrophage-colony-stimulating factor, produced mainly by T-helper cells type 2 (Th2) and innate lymphoid type 2 cells (ILC2) [4] (Figure 1). A recent study by Heaney et al. estimated that up to 80% of severe asthma demonstrate an eosinophilic phenotype at least once over longitudinal follow-up [5], a higher prevalence than previously identified. T2 inflammation is associated with increased blood, sputum, and bronchoalveolar lavage fluid eosinophils, and often elevations in fractional exhaled nitric oxide (FeNO). T2 inflammation is generally suppressed by glucocorticoids; however, a subgroup of patients with severe eosinophilic asthma have persistent eosinophilic inflammation despite the use of high-dose ICS or oral glucocorticoids [6]. T2 cytokines are therapeutic targets of several of the currently available asthma biologics. 

Most asthma biologics that are approved by the US Food and Drug Administration (FDA) target T2 inflammation with specific monoclonal antibodies to IgE, and the IL-5 and IL-4/IL-13 signaling pathways. The exception is an anti-thymic stromal lymphopoietin (TSLP) human monoclonal antibody, which is also effective among individuals with non-T2 asthma.

Omalizumab is an anti-IgE monoclonal antibody and was the first asthma biologic approved by the FDA. Omalizumab prevents the binding of IgE to its high-affinity receptor (FcER1) present on mast cells, basophils, and dendritic cells [7]. Most omalizumab studies involved patients with moderate to severe asthma, with only a few exclusively involving patients with severe asthma. In one randomized trial of 850 patients with severe uncontrolled asthma, treatment with omalizumab for 12 months led to a 25% relative reduction in asthma exacerbations compared to the placebo. All study participants had a serum IgE level of 30 to 700 IU/mL and allergic sensitization to 1+ perennial allergens [8]. 

The three FDA-approved monoclonal antibodies that target the IL-5 pathway include mepolizumab, reslizumab, and benralizumab. Mepolizumab and reslizumab are anti-IL-5 monoclonal antibodies that have been shown to reduce asthma exacerbation rates by ~50% relative to placebo when used as add-on therapy in patients with exacerbation-prone severe eosinophilic asthma [9,10]. Benralizumab is another FDA-approved biologic for the treatment of severe eosinophilic asthma. It is a humanized monoclonal antibody that depletes eosinophils and basophils by binding a subunit to the IL-5 receptor, leading to antibody-dependent cellular cytotoxicity (ADCC). Randomized trials have shown that treatment with benralizumab reduces asthma exacerbation rates and oral glucocorticoid exposure. Treatment with benralizumab has been shown to allow tapering of daily ICS therapy to “as needed” only in a majority of well-controlled patients with severe eosinophilic asthma, while also maintaining asthma control. However, a decline in forced expiratory volume in one second (FEV1) and an increase in fractional exhaled nitric oxide (FeNO) were associated with tapering of daily ICS therapy, suggesting that residual inflammation not driven by IL5 leads to decreases in lung function [11].

Dupilumab is a human monoclonal antibody that targets IL-4Rα, thereby inhibiting both IL-4 and IL-13 signaling, which share this receptor. Dupilumab has been shown to reduce asthma exacerbations and improve lung function in patients with severe uncontrolled eosinophilic asthma and oral corticosteroid-dependent asthma. Although dupilumab does not directly target eosinophils, exacerbation reduction and lung function improvement were found to be more pronounced in patients with absolute eosinophil counts of ≥150 per microliter or FeNO ≥ 25 parts per billion [12,13].

All of the currently approved asthma biologics have been proven to improve asthma-related outcomes in patients with uncontrolled asthma that meet the prescribing criteria; however, selection among the available biologic agents remains a challenge. Head-to-head trials comparing asthma biologics have not been performed and indirect comparisons show variable results [14]. Elucidation of biomarkers that can better predict responses to a given drug (e.g., elevated FeNO and blood eosinophils predict good responses to dupilumab) is needed. Clinical trials that directly compare biologic agents can potentially aid in determining which agent would provide the most robust response for individual patients, but these studies would require a considerable number of patients and be very costly without necessarily showing significant differences. Nonetheless, better information that could help inform precision medicine will ultimately decrease costs related to treatment failures.

### 2.2. Mast Cells

Mast cells are potent sources of T2 cytokines and are elevated in the lungs of patients with severe asthma. These mast cells contain both tryptase and chymase, and levels of these mediators are elevated in bronchioalveolar fluid regardless of the asthma phenotype [15]. Determining the role of mast cells in asthma is challenging due to the difficulty in isolating sufficient numbers from tissue for analysis. However, the utilization of transcriptomics has allowed for the identification of mast cell genes such as tryptase β2 (PSB2) and carboxypeptidase A3 (CPA3), both of which are highly expressed in the sputum of patients with severe asthma. Tiotiu et al. found that sputum transcriptomic signatures measured represent mast cell activity and can reflect distinct clinical phenotypes of severe asthma based on the local microenvironment. For instance, IL-33 has been associated with asthma susceptibility and can contribute to both T2 and non-T2 inflammation [16]. IL-33-mediated mast cell activation has also been shown to be associated with a neutrophilic phenotype of severe asthma [15]. Evidence of increased CPA3 and elevated urinary tetranor-PGD-M, the major urinary metabolite of mast cell prostaglandin D2 (PGD2), suggests that mast cell activation plays a pivotal role in T2-independent severe asthma [17]. Mast cell mediators also cause bronchoconstriction, mucus production, and mucosal edema and are chemotactic for key effector cells such as Th2 cells and eosinophils [18,19] (Figure 1).

### 2.3. Macrophages

Macrophage dysfunction has been proposed to be a contributing factor of exacerbations in a subset of patients with severe asthma partly due to impaired macrophage phagocytosis of microbes, resulting in bacterial-induced exacerbations [20]. A specific macrophage receptor, the MAcrophage Receptor with COllagenous structure (MARCO), is expressed on the cell surfaces of macrophages in the airways and serves an important role in the innate immune response as a scavenger receptor targeting bacterial products. Autoantibodies targeting MARCO have been identified in the airways of a severe asthma subset. Son et al. found that autoantibodies targeting MARCO may promote dysfunctional macrophage responses to bacterial pathogens, increasing the risk for infection-related exacerbations in some patients with severe asthma [21], and that most of these were neutrophilic asthma exacerbations. 

Sputum macrophages contribute to the initiation and regulation of the inflammatory response to the altered airway bacterial environment in neutrophilic asthma. Some examples of differentially expressed genes in macrophages present in neutrophilic asthma include SLAMF7, DYSF, GPR183, CSF3, PI3, and CCR7. Pathway analysis of these differentially expressed genes in neutrophilic asthma macrophages correlates with altered bacterial response, increased neutrophil recruitment, and increased expression of phagocytosis and efferocytosis [22]. This suggests an important pathophysiological role of macrophages in neutrophilic asthma.

Alternatively activated macrophages (M2) are also implicated in T2-high asthma. Specifically, M2a macrophages induced by IL-4 and IL-13 secrete chemokines, such as CCL18 and eotaxin 2, which are known to promote eosinophilic infiltration [23] (Figure 1). In addition to chemokine secretion, M2a macrophages express IL-10 and TGF-beta, which participate in airway remodeling [24].

### 2.4. Neutrophils

Compared to healthy controls and people with nonrefractory asthma, those with refractory asthma have been found to have increased airway neutrophilia [25]. Neutrophilic asthma has been associated with corticosteroid resistance, airflow limitation, and airway dysbiosis. Various studies have identified target genes that may play a role in neutrophilic inflammation in severe asthma. A specific molecular cluster in the U-BIOPRED cohort that is characterized by predominant sputum neutrophilic inflammation, inflammasome activation, and neutrophilic activation was found to have high expression of FURIN in the sputum. This finding suggests a potential link of FURIN with neutrophil activation, inflammasome, and IL-6 activation pathways [26]. Neutrophilic airway inflammation can also be driven by innate immune responses, particularly due to caspase 1 and IL1-β activity, as part of nucleotide-binding oligomerization domain leucine-rich repeat and pyrin domain-containing protein (NLRP3) inflammasome activation [27]. It is postulated that this could contribute to the steroid resistance observed in severe neutrophilic asthma.

Extracellular DNA released by activated airway neutrophils in asthma may contribute to inflammation. Extracellular DNA forms neutrophilic extracellular traps (NETs), which are important in innate immunity. However, excessive extracellular DNA and increased production of NETs can promote airway mucosal inflammation [28]. Investigators from the NHLBI Severe Asthma Research Program-3 observed that increased sputum neutrophil counts and increased sputum extracellular DNA production are associated with greater asthma severity [29]. Increased sputum extracellular DNA has also been associated with poor symptom control and increased risk of severe exacerbations [30].

### 2.5. Th17 and Innate Lymphoid Type 3 Cells

T_H_17 and innate lymphoid type 3 cells (ILC3s) produce the IL-17 family of cytokines that can trigger neutrophilic inflammation and airway remodeling in severe asthma [31] (Figure 1). IL-17A production in patients with asthma has been associated with airway hyperresponsiveness and asthma severity [32]. IL-17F level is also increased in patients with severe asthma and correlates with airway neutrophilia [33].

However, inhibition of IL17A signaling has not panned out to be the therapeutic option it had promised to be for refractory asthma. Initial animal studies suggested that treatment with anti-IL-17A could decrease pulmonary inflammation, edema, oxidative stress, and remodeling in a model of LPS-exacerbated asthma [34]. In contrast, a randomized, double-blind, placebo-controlled study utilizing a humanized anti-IL-17A monoclonal antibody, secukinumab, failed to improve asthma control in patients with severe asthma [35]. Brodalumab, a humanized mAb that binds to IL-17RA, thereby blocking the receptor and the downstream signal pathways of IL-17A, IL-17F, and other IL-17 isoforms, also did not achieve improvement in patients who were taking ICS and had inadequately controlled moderate-to-severe asthma [36]. 

## 3. Cytokines and Signaling Pathways

### 3.1. TSLP

The epithelial-derived cytokines, also called alarmins, include TSLP, IL-25, and IL-33 (Figure 1). These innate cytokines are released by airway epithelial cells in response to allergens, airborne pollutants, and viruses. Interfering with these upstream targets leads to reductions in T2 cytokines, as well as reductions in other inflammatory pathways [37]. Tezepelumab is an anti-TSLP human monoclonal antibody that has been shown to reduce the annualized rate of asthma exacerbations compared to the placebo, irrespective of absolute eosinophil counts or other markers of T2 inflammation [38]. 

### 3.2. IL-1 Receptor Family

IL-1, which exists as IL-1α and IL-1β, is a proinflammatory cytokine that is primarily produced in the lung by the airway epithelium and macrophages. IL-1α and IL-1β are part of a larger family of proinflammatory cytokines including IL-18, IL-33, and IL-36, and are essential in defense against noxious agents including particulate matter, ozone, cigarette smoke, and respiratory viruses [39].

Respiratory viruses, such as rhinovirus, are the most common triggers for asthma exacerbations. Rhinoviruses promote the release of IL-1α and IL-1β from bronchial epithelial cells and promote the production of neutrophil attractants such as IL-6 and IL-8. In a study by Schowerer et al., human bronchial epithelial cells from deceased donors were treated with IL-13 to mimic a T2-high asthmatic environment. These bronchial epithelial cells were then infected with human rhinovirus-16, which promoted the release of IL-1α, IL-6, IL-8, and CXCL10. A group of these bronchial epithelial cells were treated with an IL-1 receptor antagonist, which reduced the rhinovirus-induced expression of neutrophilic chemoattractants and MUC5B expression without changing rhinovirus infectivity and viral load. This suggests that the IL1-receptor blockade has the potential to target neutrophilic inflammation without attenuating antiviral responses [40]. 

Molecular phenotyping of sputum cells has been used to classify asthma phenotypes including eosinophilic and neutrophilic asthma [41]. It has been shown that in patients with severe asthma, there has been a high expression of genes from the IL-1 receptor family. Specifically, IL1RL1 expression has been associated with eosinophilic asthma, and NLRP3 expression has been associated with neutrophilic asthma [42]. In a murine model of severe, steroid-resistant asthma triggered by *Chlamydia* respiratory infection and ovalbumin-induced allergic airway disease, administration of anti-IL-1β antibody and NLRP3 inhibitor (MCC950) suppressed neutrophilic inflammation and reduced IL-1β production in the lung [43].

Commercially available drugs that target IL-1, including anakinra, a recombinant human IL-1 receptor antagonist, and canakinumab, a human monoclonal antibody targeting IL-1β, have been investigated in the treatment of asthma [39]. Two-phase 1/2 clinical trials evaluated the effect of anakinra on the early and late phases of allergic airway inflammation in asthma [44,45]. However, these trials were withdrawn due to risks associated with allergen challenge and anakinra treatment. A clinical trial evaluating canakinumab showed it to be effective in lowering the late asthmatic response in patients with mild asthma [46]. The availability of biomarkers that identify IL-1-high asthma would potentially allow for the utilization of these available therapeutic options, but this strategy first requires validation from supportive phase 3 clinical trials. 

### 3.3. IL-6

IL-6 is a marker of systemic inflammation, obesity, and metabolic dysfunction, and its signaling has been linked to severe asthma (Figure 1). IL-6 might induce asthmatic airway pathology through its effects on epithelial and other structural airway cells [47]. Increased levels of IL-6 and the soluble IL-6 receptor detected in the serum of severe asthma patients [48,49], as well as serum IL-6 levels, are independent predictors of future exacerbations among patients with severe asthma [50]. 

Soluble IL-6Rα amplifies IL-6 signaling bronchial epithelial tissue, and high levels of this signaling have been observed in asthma patients with low sputum eosinophils [51,52]. This finding suggests that this severe asthma subset may benefit clinically from therapeutics that target this signaling pathway, with clinical trials underway [53]. Tocilizumab is a humanized IgG1 antibody targeting the IL-6 receptor and has been used in the treatment of rheumatoid arthritis; more recently, it has also been used in treating severe COVID-19 infection [54]. There is limited data available regarding the use of tocilizumab in the treatment of severe asthma. Etsy et al. reported two cases of pediatric nonatopic severe asthma treated with tocilizumab. Both patients had an increase in FEV1, a reduction in oral corticosteroid use, and reduced IL-4 and IL-17 expression after treatment with tocilizumab [55]. 

### 3.4. CCL5

A subset of individuals with severe asthma have an overlapping Th1 and Th2 phenotype. CCL5 is a strong recruiter of Th1 cells via the CCR5 receptor and a known recruiter of eosinophils via the CCR1 receptor, making it a unique bridge for this phenotype and a novel target for asthma therapeutics in this population [56]. Gauthier et al. evaluated the role of CCL5 in tissue-resident memory T cell reactivation and found that sputum CCL5 expression correlated with T1 inflammation, but was also associated with greater fractional exhaled nitric oxide, greater blood and sputum eosinophils, and greater sputum neutrophils. Treatment with the CCR5 inhibitor maraviroc in a murine model blunted airway responsiveness and lung inflammation, suggesting an important role for CCL5 in tissue-resident memory T cell reactivation [56]. This strategy has not yet been tested in clinical trials. 

### 3.5. CC16

Club cell secretory protein 16 (CC16) is an anti-inflammatory protein secreted in large amounts in the airways, which maintains homeostasis of the airway epithelium and protects the airway from oxidative stress. Low levels of CC16 have been associated with obstructive lung diseases including asthma [57]. Low plasma CC16 has been shown to be associated with the presence of asthma with frequent symptoms from childhood to adulthood, and in children, it can be predictive of the persistence of frequent asthma symptoms into adulthood [58]. This suggests that CC16 may have a potential role in asthma risk stratification as well as targeted treatment.

### 3.6. RGS Pathway

The airway hyperresponsiveness that occurs in asthma is a result of airway smooth muscle constriction. This constriction occurs via G protein-coupled receptor (GPCR)-mediated bronchoconstrictive signaling. Regulators of G protein signaling (RGS), specifically RGS2, play a role in modulating this bronchoconstrictive signaling. *RGS2* knockout mice exhibit increased airway hyperresponsiveness and remodeling in models of asthma [59]. More recently, a study by Cardet et al. found that two *RGS2* promoter variants that associate with decreased *RGS2* expression associate with higher rates of asthma exacerbations among non-Hispanic White participants with moderate to severe asthma from the SARP3 cohort [60]. 

### 3.7. Drivers of Airway Remodeling in Asthma

Airway remodeling is a defining feature of asthma and is characterized by structural alterations in the airway, which include airway smooth muscle hypertrophy and hyperplasia, goblet cell metaplasia, angiogenesis, collagen deposition in the basement membrane, and epithelial barrier damage [61]. The airways of individuals with severe asthma have more elastin compared to healthy controls due to the effects of TNFα, IL-1β, and TGFβ on myofibroblasts [62]. Sanchez et al. found that elevated levels of IL18R1 found in the sputum of individuals with severe asthma are potentially linked to epithelial dysregulation driving fibroblast differentiation towards myofibroblasts and mucus production [63].

### 3.8. Lipid Mediators

Sputum supernatant is comprised of a mixture of lipids, proteins, pulmonary surfactant, immune cells, epithelial cells, and saliva. A U-BIOPRED study by Brandsma et al. looked at the lipid composition of induced sputum supernatant from healthy controls and asthma patients of all disease severities using lipidomics. This study found that sputum lipid phenotypes with higher levels of lipids that were cell-derived and nonendogenous to pulmonary surfactant were associated with worse asthma severity, lower lung function, and increased granulocyte counts. This finding suggests that surfactant dysregulation caused by lipids released by granulocytes recruited to the airways reduces the ability of surfactant to lower surface tension, leading to airway collapsibility and compromising its immunologic function [64].

### 3.9. Microbiome

The lung microbiota in patients with asthma is noted to be altered. Lung bacterial dysbiosis is believed to have a causative role in asthma pathogenesis [65]. This is especially true in early life, where the microbiome serves as an interface between exogenous factors and immune development. Abdel-Aziz et al. found that analysis of the oropharyngeal microbiota of children with asthma could potentially be used to define asthma clinical phenotypes based on bacterial profiles [66]. This serves as a putative novel target that may be utilized to influence asthma trajectory.

In adults with severe asthma and children with persistent wheezing, the respiratory microbiome has been noted to have less diversity and increased abundance of *Moraxella*, *Streptococcus*, and *Haemophilus* [67].

An unbiased clustering analysis performed on patients with severe asthma from the UBIOPRED cohort identified a cluster of patients with elevated sputum neutrophilic inflammation, deficiency of commensal bacterial diversity, decreased sputum macrophages, and worse lung function outcomes. The deficiency in commensal bacteria was associated with an increased risk of infection from pathogenic bacteria. The microbiome profile was noted to be stable over 12 to 18 months [68]. These findings suggest that the sputum microbiome may serve as a potential biomarker in characterizing severe asthma. 

### 3.10. Obesity 

Obese patients with asthma have been shown to have increased airway collapsibility, and consequently, a tendency to develop more airway trapping [69]. In asthma, the presence of air trapping is associated with more severe disease. A recent study by Leung et al. found that air trapping is more severe in patients with asthma who have the presence of mucus plugging and airway eosinophilia. Interestingly, obese patients were found to have less severe air trapping and less involvement of air trapping in the lower lobe segments of the lungs. This is believed to be due to the mass effect placed on lower lobe segments by central adipose tissue, causing decreased expiratory lung volumes and less susceptibility to air trapping [70]. In the study by Tattersall et al., lower paraspinal muscle density, which reflects greater muscle fat infiltration, was found to predict lung function decline only among women with asthma, suggesting that metabolic dysfunction plays a sex-dependent role in lung function decline [71].

## 4. Current Management Options of Refractory Asthma

### 4.1. Chronic Macrolide Therapy

Macrolide antibiotics have antibacterial and anti-inflammatory effects and have been noted to be beneficial for both T2 and non-T2 asthma. A large randomized, double-blind, placebo-controlled clinical study demonstrated that oral azithromycin used as add-on therapy significantly reduced asthma exacerbations and improved quality of life in patients with persistent uncontrolled asthma [72]. 

Further studies revealed that azithromycin treatment reduces key sputum cytokines associated with non-T2 asthma, such as IL-6 and IL-1β [73]. Current asthma guidelines endorse the use of low-dose azithromycin for severe asthma [74]. 

### 4.2. Treatable Traits

Identifying and managing comorbid conditions can provide significant symptom improvement in patients with severe asthma. Smoking, obesity, rhinosinusitis, obstructive sleep apnea, anxiety, and depression were found to be among the most prevalent extrapulmonary treatable traits associated with severe asthma, and managing them is an important component of comprehensive severe asthma management [75]. 

## 5. Potential New Targets of Refractory Asthma

### 5.1. Targeting Mast Cells

Masitinib is an oral tyrosine kinase inhibitor that targets mast cell activity and platelet-derived growth factor receptor (PDGFR) signaling. When compared to placebo, masitinib reduced the annualized severe asthma exacerbation rate in adults with oral corticosteroid-dependent severe asthma by 35%. This demonstrates a potential management strategy for corticosteroid-dependent severe asthma that is unrelated to the mechanisms associated with the current T2 asthma biologics [76]. Imatinib, another oral tyrosine kinase inhibitor, was tested for the management of severe refractory asthma and although it was found to reduce airway hyperresponsiveness as compared to the placebo, it was also associated with several adverse effects including muscle cramps and metabolic abnormalities, most commonly hypophosphatemia [77]. However, newer c-kit inhibitors have been shown to have a better safety profile and their positive effects in urticaria have encouraged companies to explore their role in the management of severe asthma. Clinical trials testing optimal responders to cKIT inhibition in severe asthma are underway [53]. 

### 5.2. Targeting IL-33

Astegolimab, a human IgG2 monoclonal antibody that selectively inhibits the IL-33 receptor ST2, reduced annualized asthma exacerbation rates relative to placebo in adults with severe asthma. This population included eosinophil-low adults with severe asthma [78]. In a placebo-controlled 4-arm trial, itepekimab, a monoclonal antibody against IL-33, was found to be efficacious for asthma control and improving lung function compared to the placebo. Its efficacy was similar to that of the anti-IL4/IL13 monoclonal antibody dupilumab arm, but the combination of itepekimab and dupilumab arm was not superior to either agent alone [16]. Biologics targeting the IL33 pathway remain promising therapeutic options.

### 5.3. Targeting Janus Kinases 

Several of the inflammatory pathways in asthma involve cytokines that activate Janus kinases (JAKs). JAK inhibitors have demonstrated efficacy and are clinically available for the management of inflammatory diseases including atopic dermatitis and rheumatoid arthritis [79]. JAK-dependent signaling pathways are an attractive therapeutic target for asthma, but the side effect profile is a major consideration in the development of any novel asthma therapeutic. Most of the current FDA-approved JAK inhibitors have the potential to cause systemic adverse effects, including dose-dependent myelosuppression, which is believed to be due to the inhibition of JAK2-dependent hematopoietic growth factors [79]. Recent studies have shown that inhibitors of JAK3 might prove useful in asthma [80]. In addition, both animal and human studies have demonstrated the potential of inhaled JAK inhibitors in asthma, which is promising for the benefit of lung retention and minimal systemic exposure. 

In a murine model of steroid-resistant asthma, multipotent ILC2s induced by IL-33, TSLP, and IL-2/IL-7 were found to contribute to steroid resistance. Blocking the JAK3 pathway with JAK3 inhibitor PF06651600 in this steroid-resistant murine model inhibited formation of the multipotent ILC2s in vitro and ameliorated *Alternaria*-induced asthma [80]. Findings from this study imply a role for JAK3 inhibitors in corticosteroid resistance asthma. Calbet et al. evaluated the efficacy of inhaled, selective pan-JAK inhibitor LAS194046 in a rodent model of ovalbumin-induced airway inflammation. LAS194046 was found to reduce ovalbumin-induced airway inflammation, the late-phase allergen response, and phosphorylated STAT activation [81]. The combination of LAS194046 with inhaled fluticasone propionate had a synergistic anti-inflammatory effect demonstrated by GRE gene sequence activation, mitogen-activated protein kinase phosphatase 1 (MKP1) induction, reduction in phosphoinositide 3-kinase δ (PI3Kδ) expression and activity, and a decrease in extracellular signal-regulated kinase 1/2 (ERK 1/2), P38, and JAK2/signal transducer and activator of transcription 3 (STAT3) phosphorylation [82]. Preclinical data for two inhaled JAK1 inhibitors, AZD0449 and AZD4604, have demonstrated specific JAK1 inhibition in the lung. Both compounds inhibited JAK1-dependent cytokine signaling pathways in a dose-dependent manner in human and rodent leukocytes. In an ovalbumin-induced rat model, both AZD0449 and AZD4604 demonstrated phosphorylation of STAT3, STAT5a, and STAT5b in lung tissue signifying inhibition of JAK1-dependent signaling. In this same ovalbumin-induced rat model, intrathecal administration of AZD0449 and AZD4604 inhibited eosinophilia in the lung and reduced the late asthmatic response as measured by an average enhanced pause (Penh). In a double-blind, randomized, placebo-controlled, phase 1 proof-of-activity study, the inhaled, selective small molecule JAK inhibitor GDC-0214 was found to cause a dose-dependent reduction in FeNO in patients with mild asthma at the end of a 10-day treatment period. The treatment was overall well tolerated without evidence of associated systemic toxicity [83]. The data from these studies suggest a promising role for JAK inhibition in management of refractory asthma and for providing insight into asthma pathobiology. 

### 5.4. Targeting Tumor Necrosis Factor α (TNFα)

TNFα is a proinflammatory cytokine that has increased expression in the airways of patients with asthma [84]. However, targeting TNFα has not been successful in asthma clinical trials and is associated with adverse events. In a randomized, double-blind, placebo-controlled trial, treatment with an anti-TNFα receptor antagonist, Etanercept, versus the placebo did not prevent the development of pulmonary eosinophilia after bronchoscopic allergen challenge. It was also associated with an increase in pulmonary IL-4 levels [85]. A trial that tested the efficacy of golimumab, a monoclonal antibody that targets TNF-α, in a large population of patients with uncontrolled severe asthma failed to demonstrate significant changes in lung function or reductions in severe exacerbations through week 24 of treatment compared to the placebo. An unfavorable risk-to-benefit profile led to an early discontinuation of the study after week 24. Nearly a third of patients treated with golimumab experienced serious adverse events, including one death and eight malignancies [86].

Although targeting TNFα has been found to be very effective in the treatment of some inflammatory conditions such as rheumatoid arthritis and inflammatory bowel disease [87], this mechanism has been found to be less useful than expected in severe asthma. Perhaps with better characterization of unique asthma endotypes, a subset of patients with severe asthma that respond safely to TNFα blockade may be established.

### 5.5. Targeting OX40 Ligand

OX40 ligand (OX40L) and its receptor OX40 are members of the TNF receptor superfamily. OX40L is expressed in a range of cells including dendritic cells, natural killer cells, B lymphocytes, and macrophages, and is directly mediated by TSLP [88] (Figure 1). OX40 is a costimulatory molecule transiently expressed on activated T cells. Siddiqui et al. demonstrated that OX40L and OX40 expression was increased in the lamina propria of asthma patients [89]. In a study comparing pediatric patients with asthma on inhaled corticosteroids and healthy controls, OX40L levels were higher in patients with asthma compared to healthy controls, particularly patients with steroid-resistant asthma. OX40L levels were also positively correlated with serum IgE, serum IL-6, TSLP, and eosinophil and neutrophil percentage [90].

In a double-blind, placebo-controlled trial, blockade of OX40L using a humanized OX40L monoclonal antibody failed to attenuate the early and late phase asthma response compared to placebo. Decreases in serum total IgE level and sputum level eosinophils were observed in the treatment group; however, there was no effect on airway hyperresponsiveness or blood eosinophils [91]. More recently, a murine model demonstrated that the dual blockade of OX40L and CD30L, another member of the TNF receptor superfamily, reduced eosinophilic airway inflammation and led to fewer effector memory Th2 cells accumulating in the lung without impacting the generation of regulatory T cells [92]. These data suggest that T cell costimulatory molecules may serve a role as therapeutic targets for asthma, but further investigation is warranted.

## 6. Conclusions

Severe refractory asthma is a complex and heterogeneous disease encompassing a variety of molecular pathways. Understanding the intricate molecular biologic pathways unique to severe asthma can pave the way for the development of targeted therapies using a precision medicine strategy centered around the predominant pathway. Targeted therapies using biologics have revolutionized the treatment of refractory asthma; however, all of the clinically available biologics are most effective among patients with T2-high asthma. Patients with non-T2 asthma, driven by any of the mechanisms described in this review, remain vulnerable to asthma exacerbations and progressive lung function decline due to airway remodeling. These patients stand to gain from novel targeted therapies that address the cells, mediators, and pathways described in this review. 

## Data Availability

No new data were created or analyzed in this study. Data sharing is not applicable to this article.
